# Assessment of intratumor hypoxia by integrated ^18^F-FDG PET / perfusion CT in a liver tumor model

**DOI:** 10.1371/journal.pone.0173016

**Published:** 2017-03-06

**Authors:** Yong Wang, Errol Stewart, Lise Desjardins, Jennifer Hadway, Laura Morrison, Cathie Crukley, Ting-Yim Lee

**Affiliations:** 1 Department of Radiology, Union Hospital, Tongji Medical College, Huazhong University of Science and Technology, Wuhan, Hubei, China; 2 Department of Medical Biophysics, Schulich School of Medicine & Dentistry, Western University, London, Ontario, Canada; 3 Imaging Program, Lawson Health Research Institute, London, Ontario, Canada; 4 Imaging Research Laboratories, Robarts Research Institute, London, Ontario, Canada; Beth Israel Deaconess Medical Center, UNITED STATES

## Abstract

**Objectives:**

Hypoxia in solid tumors occurs when metabolic demands in tumor cells surpass the delivery of oxygenated blood. We hypothesize that the ^18^F-fluorodeoxyglucose (^18^F-FDG) metabolism and tumor blood flow mismatch would correlate with tumor hypoxia.

**Methods:**

Liver perfusion computed tomography (CT) and ^18^F-FDG positron emission tomography (PET) imaging were performed in twelve rabbit livers implanted with VX2 carcinoma. Under CT guidance, a fiber optic probe was inserted into the tumor to measure the partial pressure of oxygen (pO_2_). Tumor blood flow (BF) and standardized uptake value (SUV) were measured to calculate flow-metabolism ratio (FMR). Tumor hypoxia was further identified using pimonidazole immunohistochemical staining. Pearson correlation analysis was performed to determine the correlation between the imaging parameters and pO_2_ and pimonidazole staining.

**Results:**

Weak correlations were found between blood volume (BV) and pO_2_ level (r = 0.425, P = 0.004), SUV and pO_2_ (r = -0.394, P = 0.007), FMR and pimonidazole staining score (r = -0.388, P = 0.031). However, there was stronger correlation between tumor FMR and pO_2_ level (r = 0.557, P < 0.001).

**Conclusions:**

FMR correlated with tumor oxygenation and pimonidazole staining suggesting it may be a potential hypoxic imaging marker in liver tumor.

## Introduction

Hypoxia, a hallmark of malignant diseases, is highly associated with an aggressive tumor phenotype and therapeutic resistance [1]. A variety of invasive and non-invasive methods have been developed to detect and monitor spatial and temporal hypoxia in tumors, including biological markers and non-invasive imaging techniques [2]. Positron emission tomography (PET) imaging has been investigated for imaging tumor hypoxia using a variety of tracers, including 18F-fluoroazomycinarabino-furanoside (18F-FMISO), 18F-fluoroazomycinarabinofuranoside (18F-FAZA) and Cu (II)-diacetyl-(N4-methylthiosemicarbazone (Cu-ATSM). However, most PET hypoxia tracers show unfavourable results in liver hypoxia imaging. The limitations of these PET tracers include low imaging contrast resulting from their relatively high lipophilicity and slow clearance characteristics; slow accumulation in hypoxic tumors; measurement of predominantly chronic rather than acute hypoxia; and absolute uptake is not purely pO_2_-dependent being confounded by changes in vascular delivery and cellular reductase expression [3]. Currently, no imaging modalities have been recommended for imaging liver tumor hypoxia in clinical practice.

Hypoxia in solid tumors is mainly due to increased metabolic demands of tumor cells surpassing the delivery of oxygen. Several studies have shown that a mismatch between blood supply and metabolism leads to aggressive tumor behaviour and more therapeutic resistance [4–6]. Moreover, there is evidence that a low-flow and high-metabolism tumor phenotype was correlated with high expression of hypoxia-inducible factor-1α (HIF-1α), which is an adaptive response to hypoxia [7]. We hypothesize that this mismatch between tumor blood flow and glucose metabolism would be significantly associated with the severity of tumor hypoxia. The increasing availability of hybrid PET- CT scanners nowadays may allow non-invasive measurements of tumor metabolism by ^18^F-FDG-PET and tumor perfusion by perfusion CT in the same imaging session. The aim of this study was to investigate the relationship between tumor perfusion and metabolism parameters as measured by PET-CT and tumor hypoxia, and determine which imaging biomarker better predicts tumor hypoxia in a liver tumor model.

## Materials and methods

Twelve male New Zealand white rabbits each weighting 3.0–3.6 kg (Charles River Laboratories in Montreal, Quebec, Canada) were used in this study. Experiments were performed in compliance with Canadian Council of Animal Care guidelines and all experimental procedures were approved by the Animal Use Subcommittee at the University of Western Ontario.

### Animal anesthesia and monitoring

The rabbit was feeding before all the studies. For each rabbit, anesthesia was induced by masking with 5% isoflurane. A marginal ear vein was catheterized and an endotracheal tube was inserted into the trachea using a laryngoscope. Anesthesia was maintained by ventilating with 2–5% isoflurane mixed with medical air at a rate of 20 breaths per minute. The animal's rectal temperature and end tidal carbon dioxide level (EtCO2) were monitored (Surgivet Advisor Vital Signs Monitor V9200, Smiths Medical, Dublin, USA). Heart rate (HR) and arterial oxygen saturation (SpO2) were continuously measured by pulse oximetry (MouseOx, Starr Life Science, Oakmont, USA). During the experimental period, animal’s rectal temperature was maintained between 38.5°C and 39.5°C with a heated water blanket. EtCO2 was maintained between 37–42 mmHg by adjusting the peak inspiratory pressure. 0.2 mL pancuronium bromide (Sandoz, Boucherville, Canada) was injected intravenously if breath-hold by turning off the ventilator was required.

### Rabbit VX2 liver tumour model

A tumor derived from implanted VX2 cells was removed from the thigh of a donor rabbit. The harvested tumor was homogenized and suspended in Hanks balanced salt solution (Sigma-Aldrich Canada, Oakville, Ontario, Canada). After anesthesia, the abdominal area of a rabbit was shaved, and 0.15–0.2 mL of the tumor suspension from the donor rabbit was directly injected into the left liver lobe of the rabbit under CT guidance to avoid large vessels. Tumor growth was monitored every 4 to 7 days after tumor implantation by contrast enhanced helical CT scanning. This scan was performed under breath hold by turning off the ventilator and after a delay of 8s from injection of 6 mL of 200 mg iodine per milliliter contrast agent (iohexol, GE healthcare, Waukesha, USA) at a rate of 1 mL per second into an ear vein. The thorax and abdomen of the rabbit were scanned with a clinical CT scanner (HD 750, GE Healthcare, Waukesha, USA) using the following technique: 60 mA, 80 kVp, 1 second gantry rotation, 5 mm slice thickness and a pitch of 0.985. When the implanted tumor had reached approximately 10–20 mm in diameter (mean diameter 16 mm ± 5, maximum diameter 23mm), integrated ^18^F-FDG PET / perfusion CT scans were performed and tumor oxygenation was measured. If the rabbit lost weight more than 20% or massive effusion was observed in the thoracic or abdominal cavity, the animal would be euthanized with an overdose of sodium pentobarbital. In this study, no animal died prior to being euthanized.

### Integrated perfusion CT / ^18^F-FDG PET

Integrated Perfusion CT/^18^F-FDG PET was performed using a hybrid 64-slice CT and PET scanner system (Discovery LS, GE Healthcare, Waukesha, USA). Imaging started with intravenous injection of 53.1 ± 3.8 MBq ^18^F-FDG through a 24G ear vein cannula. A contrast enhanced helical CT scan without breath hold was then performed to monitor the tumor size and determine the scan limits of the subsequent perfusion study. Ten minutes after the helical scan, a perfusion CT study was acquired. Five mL of 200 mg iodine per mL contrast was injected at a rate of 1 mL/sec into an ear vein at the start of the two-phase perfusion CT examination. In the first phase, the ventilator was turned off for 30 seconds; eight 2.5-mm thick sections covering the tumor were scanned continuously without breath motion. In the second phase, the ventilator was turned back on and the animal was scanned continuously for 4s (the ventilation cycle was 3s long) without breath hold every 10 seconds for 12 additional acquisitions. The scan parameters were 120 kVp, 60 mA, 1 second gantry rotation, 2.5 mm slice thickness, and total acquisition time was 150 s.

Sixty minutes after injection of the ^18^F-FDG dose and nearly 30 minutes after the perfusion CT study, PET acquisition was performed in static three-dimensional mode at two contiguous bed positions including the perfusion CT scan range, 5 minutes per bed position for a total of 10 minutes. Acquired PET data after correction for attenuation and random coincidence was reconstructed into 128 × 128 images. A total of eighty-three 4.25-mm-thick images were available covering the entire thorax and abdomen for each PET study. Prior to the PET scan, one helical CT scan with the same axial range as the PET scan was acquired with the following parameters: 140kVp, 80mA, 1 second per rotation and a pitch of 1.25. CT images were reconstructed into 5-mm-thick sections at 4.25-mm intervals for use in the attenuation correction of acquired PET data.

All imaging examinations were completed in 70.9 ± 8.1 minutes after intravenous injection of 18F-FDG.

### pO_2_ measurement

After the perfusion CT study was completed and before the ^18^F-FDG PET study, tumor oxygenation was measured using the Oxylite system (Oxford Optronix, Abingdon, United Kingdom) with a large area probe (8 mm^2^ sampling area). The probe was introduced into the tumor through a 20G catheter and advanced under CT guidance such that the tip of the oxygen probe was beyond the tumor in the adjacent normal liver tissue. The CT images acquired were used for registration with perfusion maps. Tissue oxygen partial pressure (pO_2_) was sampled by withdrawing the probe at 5.0 mm intervals. At each location, pO_2_ was recorded when the reading was stable for more than 1 minute and fluctuation range was less than 1 mmHg. Three to six measurements were taken along the needle track in each tumor. Animals were kept under anesthesia, and EtCO2 and rectal temperature were continuously monitored and maintained within normal limits during the pO_2_ measurement period.

### Perfusion parameters and PET standardized uptake value

The perfusion parameter maps were generated using CT perfusion software based on Lawrence-Lee model (GE Healthcare). Dual blood supply of liver was taken into consideration and the measurement of blood flow has been validated against the gold standard radioactive microsphere in the same liver tumor model. Details were described in previous publications [8, 9]. Image registration between the perfusion maps and axial CT acquired during oxygen probe insertion was performed using free open-source software—3D slicer 4.4 (http://www.slicer.org). Several circular regions of interests (ROIs), each of 5 mm diameter and at 5 mm interval, were used to measure perfusion measurements including blood flow (BF), blood volume (BV), mean transit time (MTT) and permeability surface (PS) along the oxygen sampling track. The ^18^F-FDG uptake within each ROI was determined from the automatically registered PET images (with respect to the perfusion CT maps) with PET-CT review software on an Advantage Window workstation (GE Healthcare). Standardized uptake value (SUV) was calculated by the following formula: SUV = mean ROI activity × body weight / Injected activity. The flow—metabolism ratio (FMR) was calculated as BF divided by SUV.

### Histopathological examination

Pimonidazole hydrochloride (Hypoxyprobe, Burlington, MA, USA) dissolved in 0.9% saline was administered intravenously at a dosage of 60 mg/kg one hour before sacrifice with an overdose of potassium chloride. The liver was excised and cut into 5-mm-thick sections in an orientation matching the CT axial images. The central 5-mm-thick section embedding the oxygen sampling track was fixed in 10% neutral buffered formalin for 24–48 hours and then phosphate buffered saline for 12 hours before being embedded in parafin. One tumor sample was excluded due to inappropriate sample preparation; therefore, eleven samples were included in the subsequent histopathological examination.

The paraffin-embedded tissue blocks were sectioned at 5 μm and deparaffinised for HE staining. For pimonidazole staining, the 5 μm sections were placed in 3% H_2_O_2_ for 10 minutes to block peroxidase activity. The sections were incubated with the FITC-labeled anti-pimonidazole antibody (Hypoxyprobe, Burlington, MA, USA) diluted 1:1000 overnight at 4°C and with secondary antibody (anti-mouse IgG, ImmPRESS kit, Vector Laboratories, Switzerland) for 40 minutes at room temperature before DAB Peroxidase Substrate Kit (3,3’—diamiobenzidine, Vector Laboratories, Switzerland) was applied.

### Semi-quantitative analysis of pimonidazole staining

**Fig 1:**
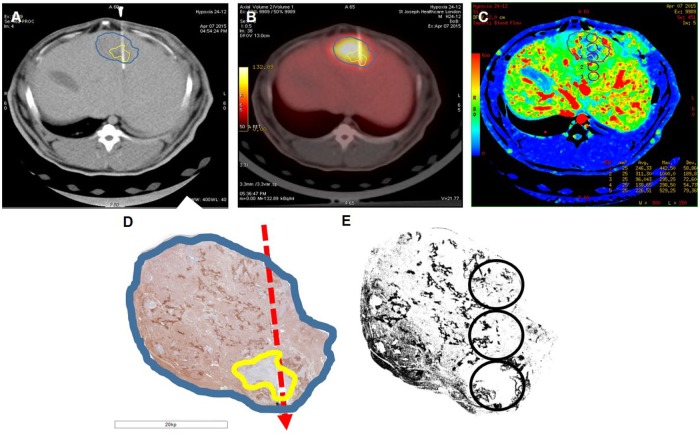
Co-registration of blood flow map from perfusion CT, PET SUV image, and pimonidazole stained section of a liver tumor. Tumor shape/edge (blue outline), necrotic area (yellow outline) and needle track (red arrow) were well-matched among the different types of images. A, Axial CT image showing the position of the oxygen probe. B, PET SUV image superimposed on CT image shown in A. C, blood flow map from perfusion CT. D, a well-matched pimonidazole stained image. E, Thresholded image of D and the circular regions along the needle track as in C.

All stained sections were scanned and digitized at a spatial resolution of 0.5 μm / pixel using TissuescopeTM 4000 scanner (Huron Technologies, Waterloo, Canada). The pimonidazole images were subsampled to the same resolution as the CT image (0.5 mm / pixel) before co-registration with the latter. Anatomic landmarks, such as tumor shape / edge, necrotic area, liver margin, blood vessel, and probe track were used for the registration. The same set of circular ROIs 5 mm in diameter and at 5 mm intervals were superimposed on each type of co-registered images: perfusion CT maps, PET SUV map, and pimonidazole stained sections (Fig 1). Pimonidazole stained area was identified and measured by setting a threshold determined with the Shanbhag algorithm [10] as implemented in ImageJ (NIH, Bethesda, MD, USA). For semi-quantitative analysis of hypoxia in each circular ROI, pimonidazole staining was scored based on the percentage of positively stained area and scored as follows: 1, 0–5%; 2, 5–15%; 3, 15–30%; 4, >30%.

### Statistical analysis

All data are reported as mean ± standard deviation. Pearson Correlation was used to determine the associations between BF, BV, MTT, PS, SUV, and FMR and pO_2_ or pimonidazole staining score as well as that between pO_2_ and pimonidazole staining score (SPSS for Windows, version 17.0; SPSS, Chicago, USA). Tumor regions were also subdivided into hypoxia or non-hypoxia groups according to pO_2_ (less or more than 10 mmHg), and the flow—metabolism ratio of each group was compared using the Mann—Whitney *U* test. A two-tailed P value of less than .05 was considered to indicate a statistically significant difference.

## Results

### pO_2_ measurements and pimonidazole staining

Tumor oxygenation measured by Oxylite showed a wide distribution ranging from 0 mmHg to 40.9 mmHg. The mean pO_2_ value in the tumor region was 9.07 ± 10.09 mmHg. In contrast, pO_2_ level in adjacent normal liver region was 27.02 ± 18.60 mmHg.

**Fig 2:**
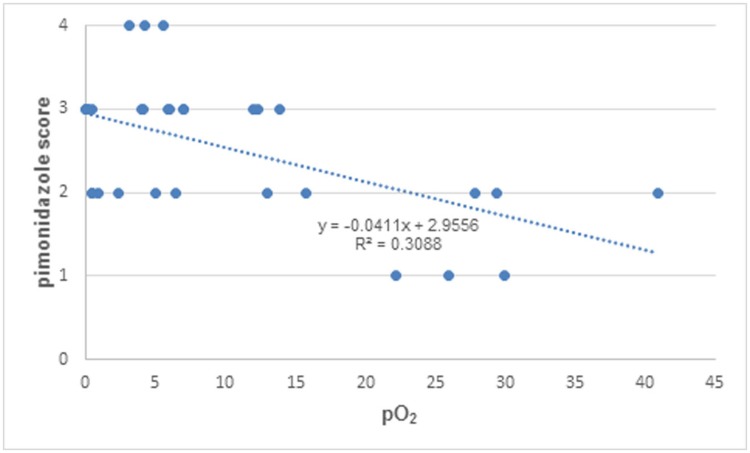
Correlation between tumor pO_2_ and pimonidazole staining score. Data from 11 rabbit VX_2_ liver tumors.

Pimonidazole staining was evident in all rabbit tumors. Positive pimonidazole staining was not only seen in tumors but also in normal liver tissues [11]. However, strong staining was only observed in tumor area peripheral to the necrotic zone. Statistically significant negative correlation was found between pimonidazole staining score and pO_2_ (r = -0.56, P < 0.001) (Fig 2).

### Correlation of perfusion parameters, glucose metabolism and pO_2_

**Fig 3:**
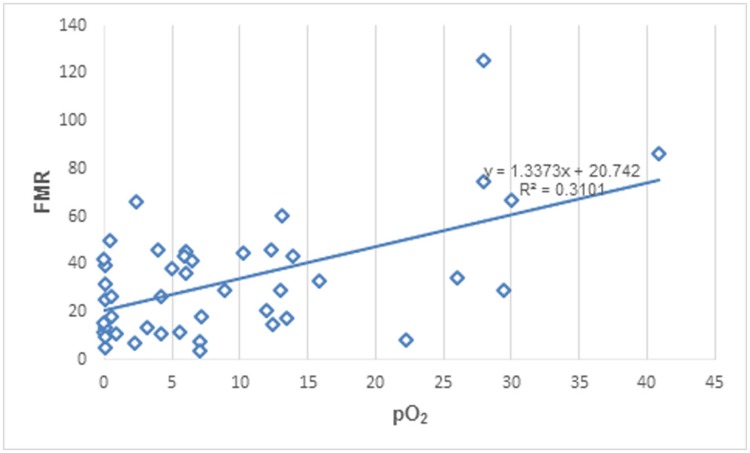
Correlation between tumor pO2 and FMR. Data from 12 rabbit VX_2_ liver tumors.

As shown in Table 1, there were no significant correlations between tumor BF, MTT, PS, ^18^F-FDG metabolism (SUV) and pO_2_ level. Significant correlations were found between BV and pO_2_ (r = 0.43, P = 0.004) and between SUV and pO_2_ level (r = -0.39, P = 0.007). Conversely, there was stronger correlation between FMR and tumor oxygenation (r = 0.56, P < 0.001) (Fig 3). According to pO_2_ level, tumor regions were subdivided into hypoxic (pO_2_ < 10mmHg) and non-hypoxic (pO_2_ ≥ 10mmHg), the mean value of FMR was 25.64 ± 16.40 in hypoxic regions, which was significantly lower than 45.98 ± 30.62 found in non-hypoxic regions (P = 0.027).

**Table 1:** Spearman rank correlation between perfusion CT parameters and pO_2_ or pimonidazole staining.

	pO_2_		Pimonidazole	
Image parameters	*r* value	*P* value	*r* value	*P* value
**BF**	0.286	0.057	-0.324	0.075
**BV**	0.425	0.004*	-0.326	0.073
**MTT**	0.136	0.373	-0.042	0.822
**PS**	0.072	0.638	-0.024	0.898
**SUV**	-0.394	0.007*	0.112	0.549
**FMR**	0.557	< 0.001*	-0.388	0.031*

Note: BF, blood flow; BV, blood volume; MTT, mean transit time; PS, permeability surface; SUV, standard uptake value; FMR, flow-metabolic ratio.

*Indicates a statistically significant correlation.

### Correlation of perfusion parameters, glucose metabolism and pimonidazole staining

**Fig 4:**
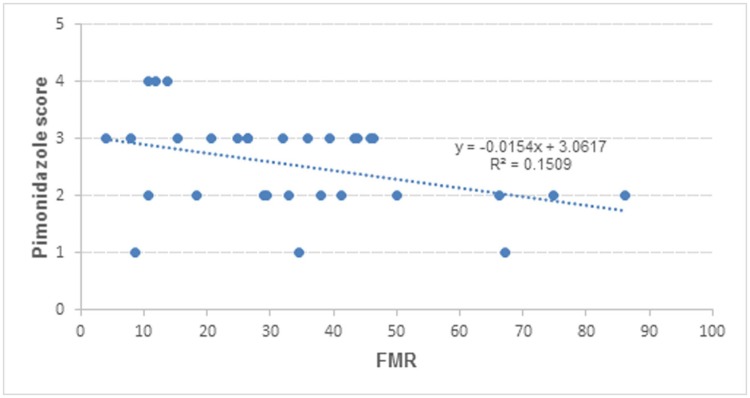
Correlation between pimonidazole staining score and FMR. Data from 11 rabbit VX_2_ liver tumors.

No correlations were observed between BF, BV, MTT, PS, and SUV and pimonidazole staining score (Table 1), but negative correlation was found between FMR and pimonidazole score (r = -0.39, P = 0.031) (Fig 4).

## Discussion

The mismatch of blood flow and metabolism in tumors as a consequence of the Warburg effect has been comprehensively reviewed by Miles and Williams [12]. Their review showed that this mismatch has been proven in a variety of tumors including brain, breast, lung, liver, colon, and head and neck. Futhermore, a number of clinical studies have shown that the mismatch is associated with tumor aggressiveness and poor survival [6, 13, 14]. Goh et al [7] using immunohistochemical staining of excised colorectal tumor samples show that low FMR was inversely correlated with high vascular endothelial growth factor (VEGF) or hypoxia-inducible factor-1 expression, suggesting that FMR may be related to hypoxia. However, as far as we know, there has been no direct comparison of FMR against pO_2_ measurement or pimonidazole staining, both are considered ‘gold’ standards for hypoxia assessment.

Currently, a variety of imaging biomarkers are under investigation for tumor hypoxia detection. Our data suggested FMR by integrated ^18^F-FDG PET / perfusion CT may be a better biomarker of hypoxia than single perfusion CT parameter (BF, BV, MTT, PS, and SUV) or SUV value of ^18^F-FDG.

In this study, only a weak correlation was found between ^18^F-FDG SUV and tumor oxygenation status (pO_2_). While ^18^F-FDG-PET has been used extensively in oncology imaging since several decades ago, the question whether ^18^F-FDG was a good surrogate for tumor hypoxia has continued to be debated. Both pre-clinical and clinical studies have investigated the relationship of SUV values with hypoxia markers and conflicting results were obtained [15–17]. Based on recent data, it appears that correlation between hypoxia and ^18^F-FDG accumulation is highly tumor type-dependent. The degree to which ^18^F-FDG uptake correlates with the severity of hypoxia may only occur in tumors with less-pronounced Warburg effect [18] or when HIF-1 activation is mainly hypoxia-driven [19]. On the other hand, although tumor oxygen delivery is dominated by blood flow, few studies have shown the correlation of perfusion CT parameters with immunohistochemical markers of hypoxia [20]. In our study, BV is the only perfusion parameter correlated with pO_2_. In the case of pimonidazole staining, it was not correlated to either perfusion CT parameters or ^18^F-FDG uptake. In normal tissue, auto-regulatory mechanisms ensures glucose metabolism and BF are tightly coupled, which is not necessarily so with tumor. The deranged vascular supply and energy metabolism within tumor cells result in inefficient oxygen delivery and use of energy substrates, a hallmark of flow metabolism mismatch. Our data suggest that this mismatch, especially in regions with low perfusion, high metabolism, is highly correlated to hypoxia based on pO_2_ measurement as well as pimonidazole staining. Thus, the FMR may be a helpful predictor for therapeutic resistance from hypoxia.

Different from previous published papers [21, 22], a statistically significant correlation between pO_2_ and pimonidazole was found. The lack of correlation in prior studies could be due to the use of a small diameter (25–30μm) oxygen needle (probe) to measure oxygen tension at a ‘single’ point in time, providing an average reading from a small ensemble of viable or necrotic cells but pimonidazole adducts, on the other hand, accumulate only in viable cells. In our study, a larger area probe (8 mm^2^ sampling area vs 19.6 mm^2^ ROIs used in the analysis of pimonidazole stained section) was employed, accordingly, the sampling volume of oxygen measurement and pimonidazole staining were more similar, reducing the inherent error in the mismatch of sampling volumes. Second, using CT guidance, the location of the oxygen probe were closely matched to the pimonidazole stained sections as well as the ^18^F-FDG SUV image (see Fig 1). Third, the number of oxygen measurement in each tumor was limited to five only to achieve a more precise measurement by averaging over a longer sampling period. The above refinements in our experimental technique likely contribute to the significant negative correlation between pimonidazole staining score and pO_2_ in our study.

As the normal liver is supplied mainly by partially deoxygenated blood from the portal vein, it is hypoxic relative to other tissues supplied by fully oxygenated blood. This explains our observation of pimonidazole staining in normal liver which is corroborated by other reported studies [11]. It raises the concern that hypoxia PET tracers, such as [18F] FMISO, could bind to normal liver and interfere with hypoxic imaging in the tumor.

Fig 3 shows that a number of tumor regions had high FMR despite the oxygen tension was close to zero. A possible explanation was that the perfusion CT study was performed before the oxygen probe insertion while the ^18^F-FDG SUV measurement was obtained after. As such, due to unavoidable tissue damage during the oxygen probe insertion, the SUV measurements of some tumor regions could be underestimated leading to an erroneously high FMR.

Several limitations of our study need to be addressed. First, as tumor hypoxia can exist both in macroscopic and microscopic scale [23], the spatial resolution of clinical PET (~ 5mm) would limit it to detecting only the macroscopic distribution of hypoxia. Second, the matching of pimonidazole stained sections to imaging (perfusion CT maps and ^18^F-FDG SUV maps) may not be perfect contributing to some of the variability in our results. Third, only one 5 μm tissue slice through (or adjacent to) the oxygen needle track was used in the analysis to simplify the analysis, which may not represent the global distribution of hypoxia. Fourth, as we discussed previous, hypoxia is highly tumor-dependent. Many factors, including tumor type, size, stage, disease site, would influence the final results. Therefore, similar investigations on different tumor types from different cell lines are needed to confirm the existence of a relationship between hypoxia and FMR. Lastly, the effective dose from the perfusion CT is relatively high ~21.3 mSv. However, this can be decreased by half if each of the ten 2^nd^ phase scans is reduced in duration from 4 to 1 s and then image registration is used to align these 2^nd^ phase images with those of the first phase [24]. Furthermore, newer compressed sensing reconstruction technique with spare view acquisition [25] can further reduce the effective dose by at least 3 times resulting in a more acceptable dose of 3.5 mSv.

## Conclusion

Our study contributes to the understanding of the relationship between functional imaging parameters and tumor hypoxia. Given the increasing availability of PET and high-end CT, FMR may be a promising image biomarker to monitor for tumor regional hypoxia, which need further investigation in various tumor types and in clinical practice.
